# Metastatic non-small cell lung cancer with BRCA2 mutation—a therapeutic challenge

**DOI:** 10.1093/omcr/omaf009

**Published:** 2025-03-28

**Authors:** Saad Salim Naviwala, Waqas Ahmed Khan, Daania Shoaib, Taha Nafees, Muzammil Shakeel, Yasmin Abdul Rashid

**Affiliations:** Department of Medical Oncology, The Aga Khan University, National Stadium Road, Karachi 74800, Pakistan; Department of Medical Oncology, The Aga Khan University, National Stadium Road, Karachi 74800, Pakistan; Department of Medical Oncology, The Aga Khan University, National Stadium Road, Karachi 74800, Pakistan; Department of Pathology, The Aga Khan University, National Stadium Road Karachi, Karachi 74800, Pakistan; Department of Radiology, The Aga Khan University, National Stadium Road Karachi, Karachi 74800, Pakistan; Department of Medical Oncology, The Aga Khan University, National Stadium Road, Karachi 74800, Pakistan

**Keywords:** non-small cell lung cancer (NSCLC), BRCA2 mutation, PARP inhibitors, adenocarcinoma, multidisciplinary team

## Abstract

Non-small cell lung cancer, specifically adenocarcinoma, is amenable to targeted therapy for genetic alterations. The role of BRCA mutations in pathogenesis and the efficacy of PARP inhibitors in these cases are still unclear. This case involves a 61-year-old male patient with a past history of Hodgkin’s Lymphoma and a heavy smoking history who developed lung adenocarcinoma and was found to have a BRCA2 mutation. Following disease progression on first-line chemotherapy and a detailed case discussion in the multidisciplinary team meeting, second-line Olaparib was started, but the patient's condition worsened rapidly, and he died from the illness. This case highlights the fact that BRCA mutations, though less common, are a potential target that should be explored further. Further large-scale studies are crucial to understanding and improving treatment outcomes for patients with BRCA-mutated NSCLC.

## Introduction

Lung cancer is the second most frequently diagnosed cancer worldwide and remains a significant global health concern. It accounted for about 2.2 million new cases and 1.8 million deaths in 2020 [1]. Since it is the organ that is most exposed to exogenous carcinogens, cancers that arise often demonstrate very high somatic mutation loads [2]. More than half of all lung adenocarcinomas have been found to carry at least one mutation. Recent advancements in identifying and targeting oncogenic driver mutations have significantly changed the standard of care and improved outcomes for non-small cell lung carcinoma (NSCLC), especially adenocarcinomas [3]. Next Generation Sequencing (NGS) is now increasingly utilized, allowing the detection of a wide range of somatic and germline mutations involved in the tumorigenesis of NSCLC [4]. Defects in the breast cancer tumor suppressor gene family (BRCA 1 & 2) are linked to several types of cancers and are well known to operate via the DNA homologous recombination and reparation mechanism. Poly Adenosine Diphosphate-Ribose Polymerase (PARP) inhibitors block DNA repair and lead to the accumulation of DNA breaks during the replication process with impaired repair and eventual cell death [5]. Although lung cancer is not typically associated with BRCA mutations, 1% of lung adenocarcinomas can harbor such alterations, with BRCA2 being the predominant subtype [6].

Here, we present the case of metastatic lung adenocarcinoma with progressive disease found to have a BRCA2 mutation on genetic testing.

## Case presentation

A 61-year-old gentleman with diabetes mellitus, hypertension, coronary artery disease, and an ECOG performance status score of 2 presented in March 2023 with a persistent cough, copious sputum, and a 20 kg weight loss over three months. He had a history of Hodgkin lymphoma treated in 1998, but no reliable records were available. He was an active smoker with a 50-pack-year history. His sister had non-Hodgkin’s lymphoma. He was of average build with normal vital signs and an unremarkable systemic examination. He brought along initial investigations from a different healthcare facility, all within reference ranges. However, a chest X-ray revealed multiple nodular densities bilaterally, with a large deposit in the right upper lobe raising a suspicion of malignancy.

**Figure 1:**
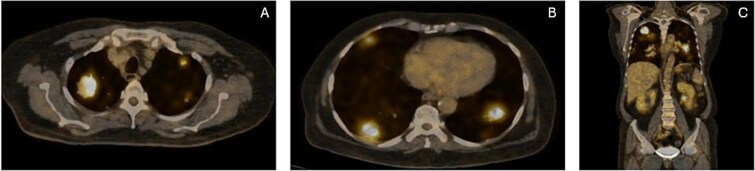
(**A**, **B** & **C**) Axial and coronal sections on the PET scan revealing bilateral pulmonary deposits.

**Figure 2:**
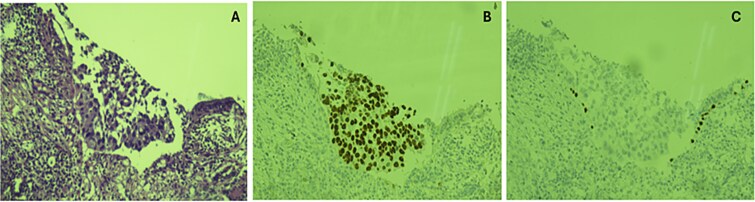
(**A**) Section showing nest of neoplastic cells with pleomorphic nuclei and eosinophilic cytoplasm. (B) TTF 1 showing nuclear positivity. (C) Lack of expression of p40 in tumor cells.

**Figure 3:**
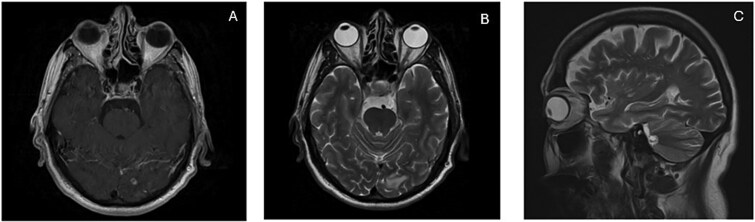
(**A** & **B**) MRI brain with and without contrast demonstrating occipital lobe deposit with surrounding edema. (C) Progressive disease with new deposit at cerebellar vermis.

Based on this, he was advised further diagnostic workup. A PET scan demonstrated multiple hypermetabolic lesions in both lung fields, with the largest in the right upper lobe having an SUVmax of 10.1, and regional lymphadenopathy (Fig. 1). An image-guided biopsy of the right upper lobe mass was carried out, and histopathology revealed adenocarcinoma of the lung (TTF-1 positive, p40 negative), as demonstrated in Fig. 2. He was advised next-generation sequencing (NGS) at diagnosis, but due to affordability issues, we had to go for a limited panel of EGFR and ALK testing only, both of which turned out to be negative. Staging was completed with an MRI brain with contrast, which revealed a solitary occipital lobe deposit measuring 6.5 × 4.2 mm, with surrounding vasogenic edema (Fig. 3).

**Figure 4:**
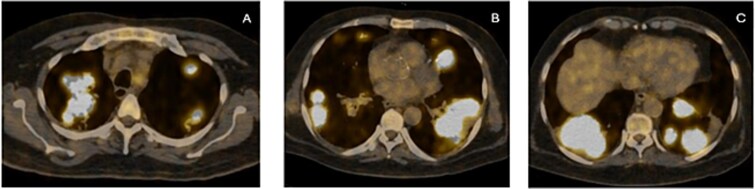
(**A**, **B** & **C**) Axial sections on PET showing gross disease progression after first-line treatment.

As first-line therapy, he was started on a combination of carboplatin (AUC 4) and pemetrexed 400 mg/m^2^. A PET scan after four cycles showed partial metabolic response, while MRI brain showed near resolution of the cerebral deposit. After completing six cycles of the combination regimen, he was switched to pemetrexed maintenance at 500 mg/m^2^. Disease assessment scans after two cycles of maintenance therapy revealed progression in the primary tumor along with new hepatic and brain deposits (Fig. 4). He underwent stereotactic radiosurgery for the brain deposit. While awaiting the report of next-generation sequencing (NGS) sent on liquid biopsy, he was started on carboplatin AUC 2 and paclitaxel 80 mg/m^2^ every week. FoundationOne NGS testing revealed a BRCA2 N1544fs*24 genomic alteration.

The multidisciplinary team discussed further care, and he was started on Olaparib 300 mg twice daily. However, his condition worsened rapidly, and he was hospitalized with pleural effusion and hypoxic respiratory failure. He was eventually transitioned to best supportive care and died in October 2023.

## Discussion

Over the past decade, notable advances have been observed in managing lung cancer, particularly non-small cell lung cancer (NSCLC). Despite significant improvements, the all-stage five-year survival rate is 23%, and less than 10% for metastatic disease at presentation [7]. Genetic alterations, including but not limited to EGFR, KRAS, ALK, MET, and ROS1, are crucial for selecting appropriate treatments, understanding resistance, and have shown promising outcomes with targeted therapy [8].

BRCA1 and BRCA2 tumor suppressor genes are primarily linked with hereditary breast and ovarian cancers and with other malignancies to a lesser extent. BRCA mutations have diagnostic and therapeutic applications in breast and ovarian cancers, including genetic testing, improved screening, and targeted therapies such as PARP inhibitors. However, the association and impact of BRCA mutations in lung cancer, particularly NSCLC, have not been as comprehensively studied [9]. Several case reports have documented varied responses in patients receiving PARP inhibitors for lung cancer with BRCA mutations. A retrospective study by Fang et al. showed that Chinese patients with BRCA1/BRCA2 mutations had a median progression-free survival (PFS) of 6 months with PARP inhibitors after multiple lines of therapy [10].

In our report, the patient did not receive therapy targeting the BRCA mutation beyond two weeks due to a worsening condition and thus was not assessed for response. Although data are limited, PARP inhibitors appear to be a viable option for patients who have progressed on or are unable to receive chemotherapy and do not have other targetable alterations aside from a BRCA mutation. Several case reports show a PFS of 6 to 10 months using PARP inhibitors in this setting.

Further research into the implications of BRCA mutations and the response to therapies such as PARP inhibitors and platinum-based chemotherapy in lung cancer is essential to broaden the therapeutic options and potentially improve patient outcomes. Additionally, it is crucial to determine whether somatic BRCA mutations identified in lung cancer warrant genetic counselling, reflex testing for germline mutations, and family screening to ensure comprehensive patient management and preventive care for relatives.
